# Type III pleuropulmonary blastoma in a 7-month-old female baby with impending respiratory failure: a case report

**DOI:** 10.1186/1752-1947-8-221

**Published:** 2014-06-23

**Authors:** Agnese Castro, Chiara Franzonello, Salvatore Leonardi, Andrea Di Cataldo, Enrico Potenza, Gaetano Magro, Giovanni A Rossi, Mario La Rosa

**Affiliations:** 1 Department of Pediatrics, University of Catania, Catania, Italy; 2 Operating Unit of Thoracic Surgery, Garibaldi-Nesima Hospital, Catania, Italy; 3 Section of Anatomic Pathology, Department G.F. Ingrassia, University of Catania, Catania, Italy; 4 Pediatric Pulmonology and Allergy Unit, Institute of Giannina Gaslini, Genoa, Italy

**Keywords:** Pediatric tumors, Respiratory failure, Thoracic CT scan

## Abstract

**Introduction:**

Pleuropulmonary blastoma is a very rare, aggressive, embryonal pulmonary neoplasm which mostly affects children under the age of 5. According to the histopathological features, three subtypes of pleuropulmonary blastoma have been recognized: type I (purely cystic), type II (grossly visible cystic and solid elements) and type III (purely solid). Characteristics of type I and type II blastoma allow an earlier diagnosis compared with type III. Here we present a case report of an unusual presentation of type III pleuropulmonary blastoma.

**Case presentation:**

We describe the case of a 7-month-old female baby of Italian mother and Kurdish father who was diagnosed with type III pleuropulmonary blastoma, which entirely occupied her right hemithorax.

**Conclusions:**

The reported case is an unusual presentation because type III pleuropulmonary blastoma typically occurs in older children. The complete re-expansion of her residual, previously totally compressed, right lung observed immediately after the resection of the lesion suggests an atypical rapid growth of this embryonal tumor in the late phase of gestation or after delivery. This case report suggests that, in addition to other childhood tumors, type III pleuropulmonary blastoma should be included in the differential diagnosis of solid nonhomogeneous thoracic large masses, compressing the mediastinal and chest wall structures in infants. This is an original case report of interest for several specialities such us pediatrics, radiology, surgery and oncology.

## Introduction

Pleuropulmonary blastoma (PPB) is a rare malignant embryonal tumor of the lungs that appears to arise during organ development and is usually diagnosed in the pediatric population under the age of 5 [1,2]. The formation of the tumor seems to begin as air-filled cysts with primitive mesenchymal cells beneath an intact benign-appearing epithelium (type I PPB). In the following stages, the mesenchymal cells overgrow the delicate septa producing a cystic and solid or exclusively solid sarcomatous neoplasm (types II and III PPB, respectively) [1-5]. The progression from type I to type II or type III PPB has been pathologically documented and observed in children diagnosed with type II or type III PPB who had chest radiographs showing lung cysts preceding the diagnosis of PPB [3-8]. The evolution from a relatively simple multicystic neoplasm, characterized by a restricted population of cells with rhabdomyoblastic differentiation, to a large solid neoplasm filling the thoracic cavity is reflective of a natural biological progression and explains the correlation between the pathological type with both age at the diagnosis and patient outcome [1,4]. Indeed, type I PPB is detected in infants (median diagnosis age, 9 to 10 months) and has a favorable prognosis with 85 to 90% overall survival [3,4]. In contrast, the more aggressive types II and III PPB are typically diagnosed in older children (median diagnosis ages, 34 to 36 and 42 to 44 months, respectively) [1,3,4] and have an overall survival estimates of 60% and 45%, respectively [1,4]. Distant metastases seem to occur only in type II or type III PPB, affecting the brain/spinal cord or bone [1,9].

Since type I PPB may recur after treatment as a type II or type III PPB and has a low recovery rate, it is vital to diagnose and treat the affected children at an early stage (type I PPB) and age, when the lesion is limited and most curable [1,4]. Unfortunately, as the initial presentation can be subtle, being characterized by a series of recurrent respiratory tract infections associated with slowly progressive shortness of breath, an early diagnosis is incidental in most cases [1-3]. Here we describe the case of a large type III PPB occupying the whole right hemithorax that had the unusual characteristic of being diagnosed in a 7-month-old female baby who, due to the size of the tumor, was hospitalized because of impending respiratory failure.

## Case presentation

**Figure 1:**
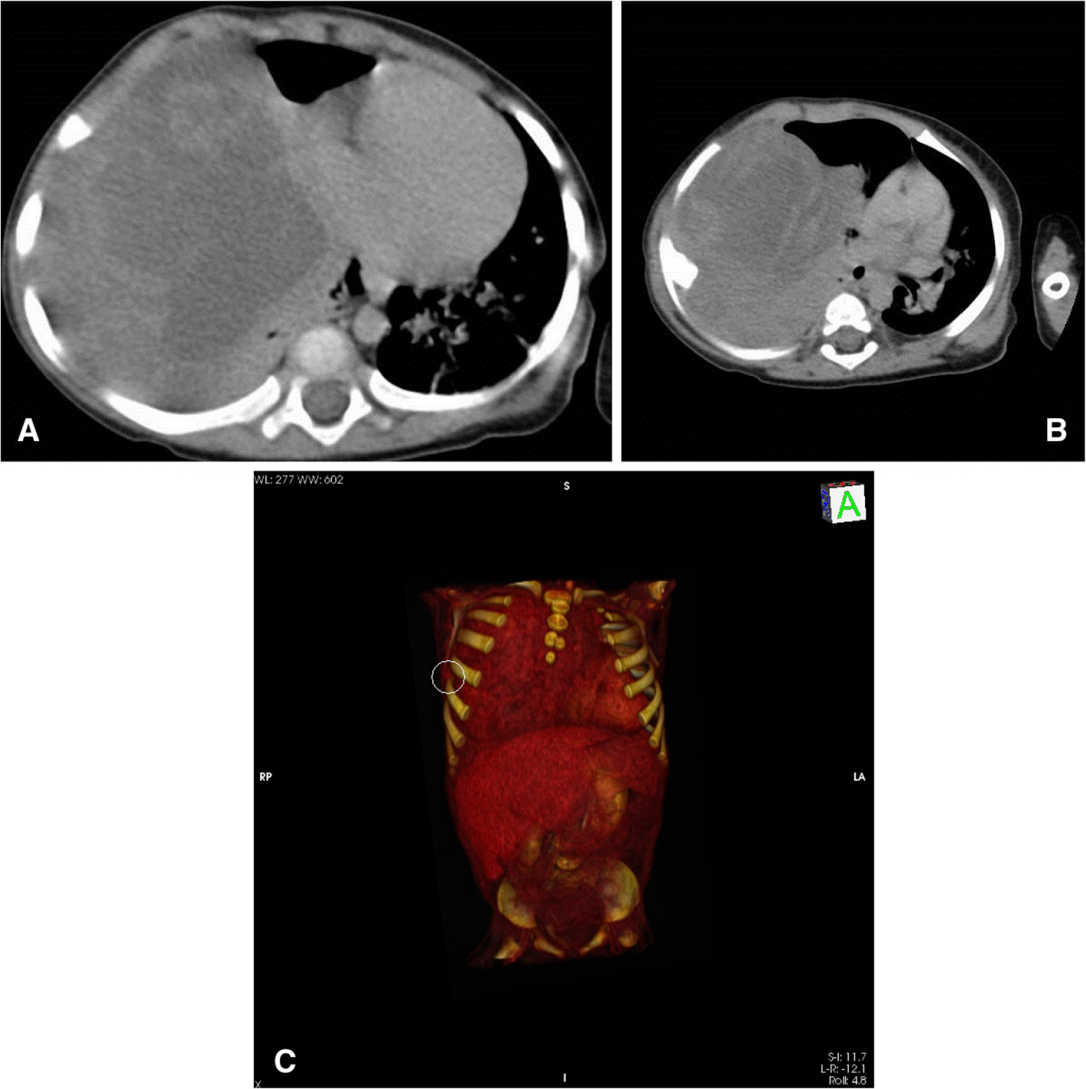
**Computed tomography scanning of the thorax.** Demonstrating the presence of an enormous mass, causing a tracheal displacement with complete collapse of the lung parenchyma **(A)** with structural alteration in the 6th right coastal arc, along the midaxillary line **(B)** better demonstrated by the three-dimensional reconstruction **(C)**.

**Figure 2:**
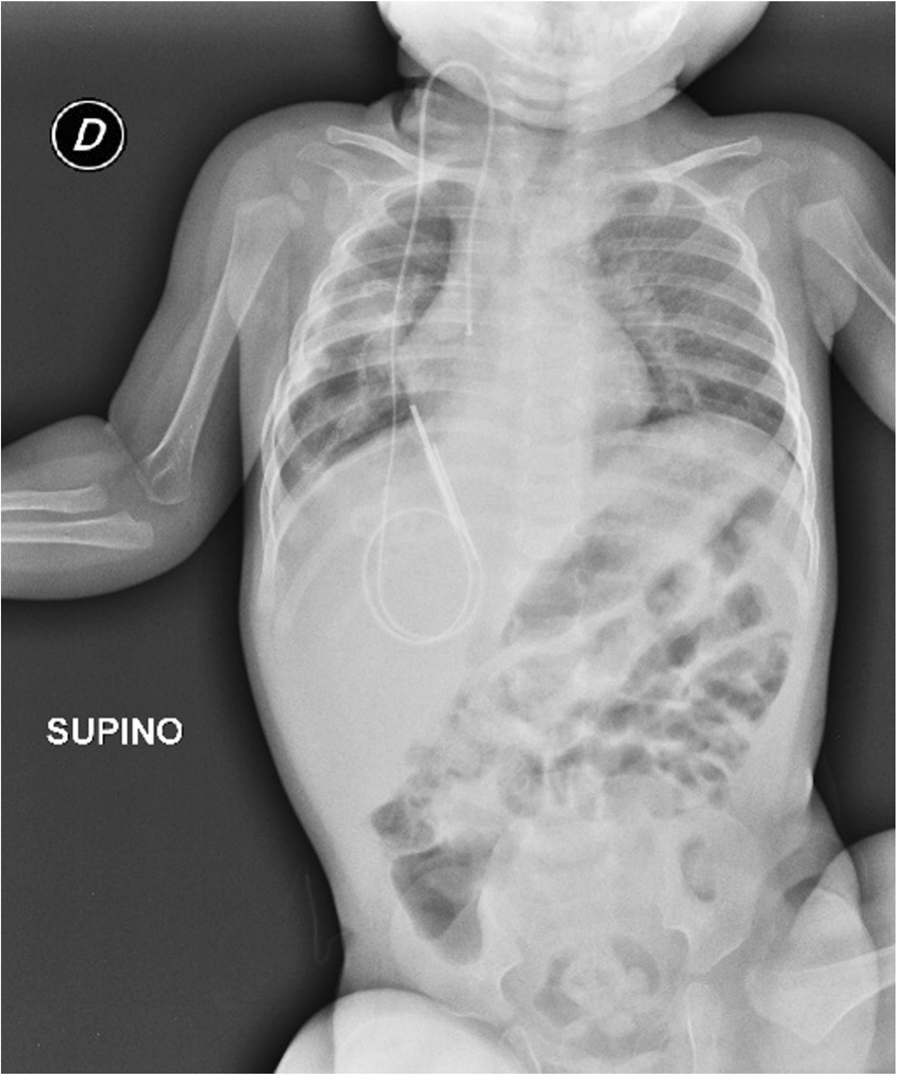
Chest radiography after the surgical removal of the intrathoracic tumor demonstrating a complete re-expansion of the right lung.

**Figure 3:**
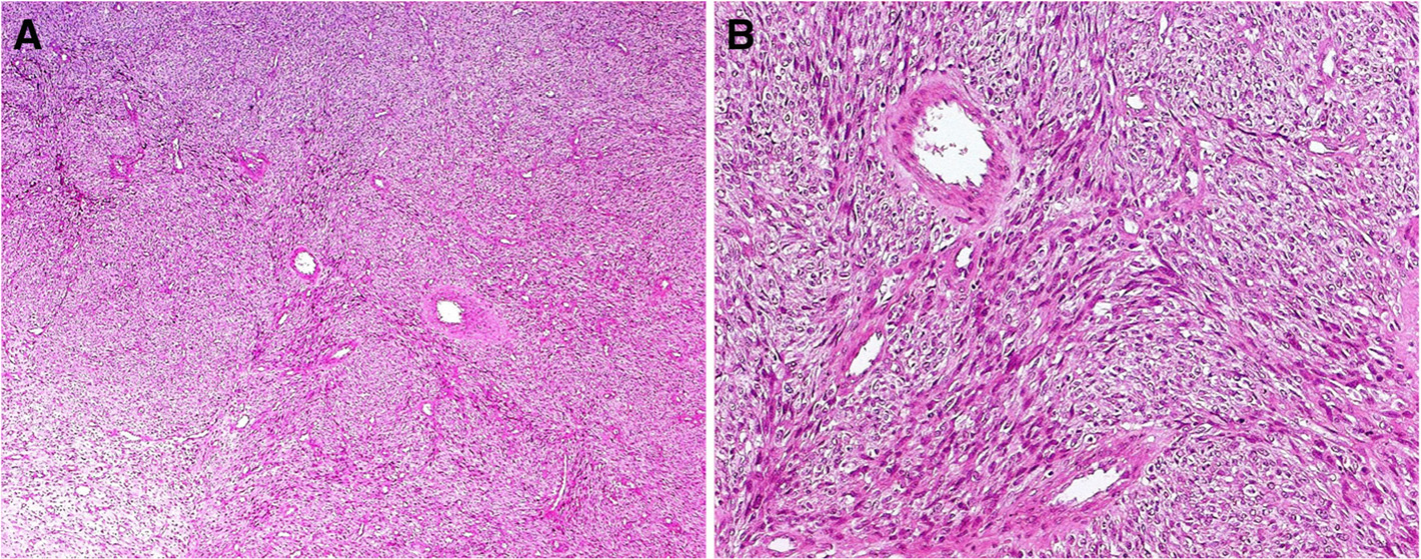
**Light microscopy picture of a specimen of the resected mass. A**: the tumor is characterized by myxoid and fibrous-like stroma, showing large areas of necrosis (hematoxylin and eosin staining, original magnification ×100). **B**: at higher magnification the tumor was constituted by blastomatous areas, containing epithelioid cells, and sarcomatous areas, containing fusiform cells (hematoxylin and eosin staining, original magnification ×250).

A 7-month-old female baby of Italian mother and Kurdish father was referred to our Pediatric Department for rapidly worsening dyspnea leading to severe shortness of breath. Respiratory symptoms, occurring intermittently in her first 2 to 3 months of life during upper respiratory tract infection, progressively worsened in length and severity and were subsequently associated with anorexia and failure to thrive. On admission, she appeared dyspneic and hypoxic and needed a high-level of oxygen (O_2_) supplementation (an inspired fraction of O_2_=5L/minute) to achieve an O_2_ saturation of 95%. Her blood pressure was 50/90mmHg, heart rate 135 beats per minute and body temperature 36.5°C. An absence of breath sounds in her right hemithorax was perceived during her chest examination. Her white blood cells were 19.23×10^3^/mm^3^ (neutrophils 62.6% and lymphocytes 27%), hematocrit 34.5%, mean corpuscular hemoglobin concentration 30.7g/dL, platelet counts 695×10^3^/mm^3^, reticulocytes 182×10^9^/L. Values lower than normal levels were detected for total proteins (5.3g/dL), albumin (2.8g/dL), iron (17μg/dL), immunoglobulin (Ig) A (30mg/dL) and IgG (625mg/dL), whereas her erythrocyte sedimentation rate (44mm/hour) and C-reactive protein (5.05mg/dL; normal range <0.80mg/dL) were elevated.Conventional chest radiography demonstrated a complete opacification of her right hemithorax with contralateral midline shift. Computed tomography (CT) scanning was performed and showed the presence of an enormous mass resulting in the contralateral displacement of her trachea, with total collapse of right lung parenchyma (Figure 1A) and compression of her chest wall tissues, causing a structural alteration of her 6th right costal arc along the midaxillary line (Figures 1B and 1C). Furthermore, enlarged lymph nodes were detected in both in her mediastinum and her right axillary region. A surgical complete resection of the mass was performed by sternotomy, which resulted in the re-expansion of her right lung, previously compressed and crouched on her mediastinum (Figure 2). The parietal pleura and her anterior mediastinum were not involved and no pleural effusion was detected. The tumor appeared to be a solid greyish-white mass, 8.5×9×5cm in size, partially covered by a thin capsule with hemorrhagic areas below. On histological examination, there was a myxoid and fibrous-like stroma showing large areas of necrosis (Figure 3A). At higher magnification the tumor was constituted by blastomatous areas (containing epithelioid cells) and sarcomatous areas (containing fusiform cells; Figure 3B). The growing pattern was fibrosarcomatous dermatofibrosarcoma protuberans-like, with a high mitotic count (30/10 high-power field) and diffused positivity for vimentin; heterogeneous positivity for S-100 protein and focal positivity for CD56 were noticed. The morphological and immunohistochemical features of the resected mass suggested the diagnosis of PPB type III. Brain magnetic resonance imaging, liver CT and skeletal scintigraphy did not reveal any secondary localization. She received adjuvant chemotherapy, which included an intensive combined therapy of ifosfamide, vincristine, actinomycin D and doxorubicin for 25 weeks (IVADo). Due to the radical resection and the absence of metastasis, postoperative irradiation was not performed.

## Conclusions

The occurrence of a type III PPB occupying the whole right hemithorax in a 7-month-old female baby is an unusual finding, suggesting an atypical rapid progression of this embryonal tumor in the late phase of gestation or after delivery. In our patient, nonspecific symptoms such as cough and mild dyspnea were noticed during infectious processes of the respiratory tract occurring 4 to 5 months before the final diagnosis. These symptoms rapidly worsened leading to respiratory failure. Presurgical imaging techniques clarified the features and the extension of the mass yet the specific diagnosis was based on histological examination. The radical resection of the mass and the absence of metastasis are favorable prognostic factors. Indeed, in addition to the histological type, the survival rate depends on the local recurrence and the risk of secondary localization in the lungs, thoracic structures, brain or spinal cord [8]. From a clinical prospective, the present case report indicates that, in addition to other childhood tumors such as rhabdomyosarcoma, neuroblastoma and Ewing’s sarcoma tumors family, type III PPB should be included in the differential diagnosis of solid nonhomogeneous thoracic large masses, compressing the mediastinal and chest wall structures also in infants [1,3-6,8-10].

## Consent

Written informed consent was obtained from the parents of the patient for publication of this case report and any accompanying images. A copy of the written consent is available for review by the Editor-in-Chief of this journal.

## Abbreviations

CT: Computed tomography; O_2_: Oxygen; PPB: Pleuropulmonary blastoma; IgA: Immunoglobulin A; IgG: Immunoglobulin G; CD56: Cluster of differentiation 56; IVADo: Ifosfamide, vincristine, actinomycin D and doxorubicin; 3D: Three dimensional.

## Competing interests

All authors declare that they have no competing interests.

## Authors’ contributions

ADC, EP, GM contributed to acquisition of data. AC, CF, SL, GAR and MLR drafted the report and all authors contributed to revision of the report. All authors read and approved the final manuscript.
